# When a Seemingly Harmless Prescription Turns into Toxicity

**DOI:** 10.1155/2018/9724390

**Published:** 2018-09-16

**Authors:** Zurab Azmaiparashvili, Kevin Bryan Lo, Nawal Habib, Annie Hsieh

**Affiliations:** ^1^Department of Medicine, Albert Einstein Medical Center, Philadelphia, PA, USA; ^2^Department of Neurology, Albert Einstein Medical Center, Philadelphia, PA, USA

## Abstract

Valacyclovir neurotoxicity is commonly seen in the elderly and those with impaired renal function. Differential diagnosis can be challenging as a myriad of medical conditions, including herpes zoster virus associated encephalitis, may present in a similar fashion. We present a case of a 71-year-old male who presented with altered mental status in the setting of recent herpes zoster eruption. His condition was attributed to valacyclovir neurotoxicity, and initiation of appropriate supportive therapy was met with complete resolution of symptoms and normalization of cognitive function.

## 1. Introduction

Even though valacyclovir is generally well tolerated, prescribers should be aware of its potential neurotoxicity, especially in the setting of acute or chronic impairment in renal function. Since the first case report by Linssen-Schuurmans et al. in 1998 [1], various case reports, case series, and reviews have been published; however, valacyclovir neurotoxicity remains a frequently overlooked problem which may lead to significant morbidity and adverse patient outcomes.

## 2. Case Presentation

A 71-year-old African American male with past medical history significant for liver cirrhosis secondary to chronic hepatitis C infection status after orthotopic liver transplantation, end-stage renal disease secondary to hypertensive nephropathy, peripheral vascular disease status after right above-knee amputation, and seizure disorder presented to the emergency department with altered mental status. Prior to change in mental status, the patient was fully alert and oriented. Altered mental status was described as psychomotor retardation and absence of verbal response to questions. Nursing home records showed that the patient had a recent onset of shingles and was started on valacyclovir by his primary care physician three days prior to presentation. He missed his usual hemodialysis session on the day of presentation in view of change in mental status. There was no reported fever, headache, or convulsions. There was no history of illicit drug or alcohol use. Except for valacyclovir, he has not been receiving any new prescription or over-the-counter medication.

**Figure 1:**
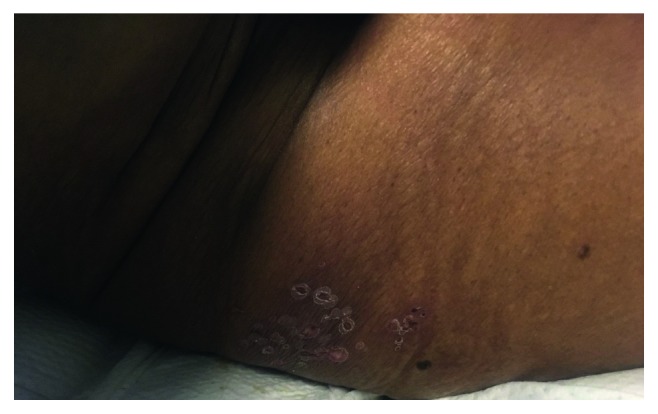
Crusted vesicles on an erythematous base over the lower back in S1 dermatomal distribution.

The patient was afebrile on admission with the normal vital signs, except for elevated blood pressure of 176/85 mmHg. On neurologic examination, the patient appeared alert and disoriented. There were no meningeal signs or focal neurologic deficits. Skin examination showed crusted vesicles on an erythematous base over the lower back in S1 dermatomal distribution, consistent with the reported history of shingles (Figure 1). Cardiovascular, respiratory, and abdominal examinations were within the normal limits.

Complete blood count revealed hemoglobin concentration of 10.0 g/dL, white blood cell count of 2,770/*µ*L, and platelet count of 201,000/*µ*L. Liver function tests were within the normal limits, while serum blood urea nitrogen and creatinine were 37 mg/dL and 6.3 mg/dL, respectively. Serum ammonia 30.8 *µ*mol/L (normal range: 18.0–72.0 *µ*mol/L). There were no significant electrolyte derangements or metabolic acidosis (Table 1). CT head with and without contrast and MRI brain showed no acute intracranial abnormalities.

**Figure 2:**
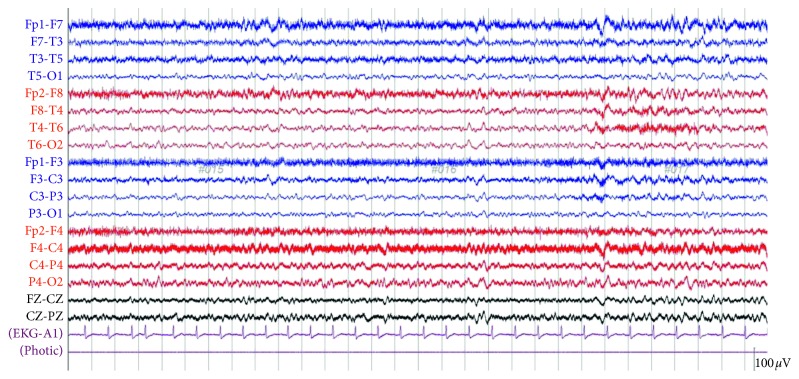
An electroencephalogram (EEG) showing bilateral diffuse slowing with theta activities with no epileptiform potentials or seizures.

Further review of nursing home records showed that the patient was prescribed valacyclovir at 1 g three times a day, which is significantly higher than the recommended dose of 500 mg daily in the presence of end-stage renal disease. At this point of time, valacyclovir was discontinued. Electroencephalogram (EEG) was performed, showing generalized slowing consistent with toxic-metabolic insult (Figure 2). Lumbar puncture was deferred as there was low suspicion for central nervous system infection, considering the absence of suggestive clinical features or imaging findings. The patient received two consecutive sessions of hemodialysis, and his mental status returned to baseline within three days of hospital admission. Together, these findings suggested valacyclovir neurotoxicity as the underlying etiology for the current presentation.

## 3. Discussion

Valacyclovir is a prodrug metabolized to acyclovir in the liver, 90 percent of which is subsequently excreted in the urine [2]. As a result, acyclovir pharmacokinetics may be significantly affected by renal impairment, leading to higher medication levels and possible toxicity. In fact, over 85 percent of cases of valacyclovir or acyclovir neurotoxicity are associated with varying degrees of renal impairment, including dialysis-dependent end-stage renal disease [2]. Age has been described as an additional risk factor, with over 80 percent of cases being reported in patients of 60 years of age and above [2]. Although neurotoxicity has also been described in patients with preserved renal function [3, 4], acyclovir has the propensity to aggravate or trigger neurotoxicity by de novo impairment of renal function through tubular precipitation and acute tubulointerstitial nephritis [5].

Symptoms of neurotoxicity typically begin within one to three days of starting the medication [6]. Disturbances in the level of consciousness and confusion are the most frequently reported symptoms, followed by disturbances of perception, including hallucinations [2]. Less commonly, neurotoxicity may manifest as ataxia, dysarthria, myoclonus, or rhabdomyolysis and in the most severe cases as seizures, coma, and death [2, 7]. Depending on the degree of renal impairment and frequency of hemodialysis, symptoms usually resolve within a week of discontinuation of the medication [6].

A proposed mechanism of valacyclovir and acyclovir neurotoxicity is through the inhibition of DNA polymerase, secondarily affecting mitochondrial DNA synthesis and leading to cellular dysfunction and subsequent neurotoxicity [8].

Valacyclovir neurotoxicity is a clinical diagnosis with a high index of suspicion based on accurate history and physical examination, and careful review of associated risk factors. Acyclovir levels may be obtained from the blood, serum, cerebrospinal fluid, or urine, based on availability; however, levels have not been shown to correlate with clinical presentation [2, 7]. Importantly, other important causes of altered mental status should be ruled out, including nonconvulsive seizures and central nervous system infections. In particular, distinction should be made between valacyclovir neurotoxicity and herpes zoster virus associated encephalitis as failure to do so may have devastating effects, especially in immunocompromised hosts, such as presented in our case. Herpes zoster virus associated encephalitis similarly presents within one to two weeks from the onset of the vesicular skin eruption with symptoms of somnolence and confusion, although headache and fever are more common. Trigeminal or ophthalmic nerve involvement or disseminated zoster infection may also point to the possibility of herpes zoster virus associated encephalitis [9]. Imaging findings, specific viral titers and electroencephalogram may be helpful in diagnosis, however, none of the tests are consistently positive and results need to be interpreted in the context of clinical presentation [9]. Considering these facts, our case presents a diagnostic dilemma, as empirically treating herpes zoster virus associated encephalitis may potentially worsen valacyclovir neurotoxicity. On the other hand, leaving herpes zoster virus associated encephalitis in an immunocompromised host untreated is likely to lead to dire consequences.

Treatment of valacyclovir neurotoxicity is supportive, including discontinuation of the culprit medication. Additionally, hemodialysis may shorten the duration of symptoms as approximately 40 to 50 percent of the drug is cleared in a 4-hour hemodialysis session [7, 10]. There are limited data to support intensification of peritoneal dialysis regimen for mitigation of neurologic symptoms [11]. Of note, failure to improve after a few sessions of hemodialysis should prompt search for other potential etiologies, such as viral encephalitis, which usually takes longer to resolve [9].

## 4. Conclusion

This case once again demonstrates the continued need for increased awareness of valacyclovir neurotoxicity, especially in the subset of patients with increased risk, including the elderly and those with acute or chronic impairment in renal function. Differential diagnoses should be considered including seizures, other central nervous system depressant medications, and viral encephalitis. There is also the need for introduction of improved patient safety checkpoints in current outpatient prescription practices.

## Figures and Tables

**Table 1:** Patient's laboratory results on admission.

White blood cell count (/*μ*L)	2,770
Hemoglobin (g/dL)	10
Platelet count (/*μ*L)	201,000
Sodium (mEq/L)	135
Potassium (mEq/L)	4.7
Chloride (mEq/L)	101
HCO_3_^−^ (mmol/L)	18
Blood urea nitrogen (mg/dL)	37
Serum creatinine (mg/dL)	6.3
Random blood glucose (mg/dL)	111
Alkaline phosphatase (IU/L)	88
Total bilirubin (mg/dL)	0.7
Direct bilirubin (mg/dL)	0.2
Aspartate aminotransferase (U/L)	17
Alanine aminotransferase (U/L)	7
Total protein (g/dL)	6.6
Albumin (g/dL)	3.8
PT(s)/INR	13.8/1.1
Venous pH	7.38
Venous CO_2_ (mmHg)	34
Serum ammonia (*µ*mol/L)	30.8

## References

[1] Linssen-Schuurmans C. D., van Kan E. J. M., Feith G. W., Uges D. R. A. (1998). Neurotoxicity caused by valacyclovir in a patient on hemodialysis. *Therapeutic Drug Monitoring*.

[2] Asahi T., Tsutsui M., Wakasugi M. (2009). Valacyclovir neurotoxicity: clinical experience and review of the literature. *European Journal of Neurology*.

[3] Yoshimura T., Kawasaki T., Shirota A., Saeki M., Okada Y., Okada H. (2018). Valacyclovir-induced neurotoxicity in a patient with a preserved renal function. *Internal Medicine*.

[4] Vander T., Medvedovsky M., Herishanu Y. (2003). Encephalopathy induced by oral acyclovir in a patient with normal renal function. *Journal of Infection*.

[5] Rashed A., Azadeh B., AbuRomeh S. H. (1990). Acyclovir-induced acute tubulo-interstitial nephritis. *Nephron*.

[6] Haefeli W. E., Schoenenberger R. A. Z., Weiss P., Ritz R. F. (1993). Acyclovir-induced neurotoxicity: concentration-side effect relationship in acyclovir overdose. *American Journal of Medicine*.

[7] Huguenel C., Felton D., Bruccoleri R., Salhanick S. (2015). Case files of the Harvard medical toxicology fellowship: valacyclovir neurotoxicity and unintentional overdose. *Journal of Medical Toxicology*.

[8] Lewis W., Dalakas M. C. (1995). Mitochondrial toxicity of antiviral drugs. *Nature Medicine*.

[9] Hughes B. A., Kimmel D. W., Aksamit A. J. (1993). Herpes zoster-associated meningoencephalitis in patients with systemic cancer. *Mayo Clinic Proceedings*.

[10] Kambhampati G., Pakkivenkata U., Kazory A. (2011). Valacyclovir neurotoxicity can be effectively managed by hemodialysis. *European Journal of Neurology*.

[11] Prasad B., McIsaac M., Toppings J. (2017). Valacyclovir-associated neurotoxicity treated with intensification of peritoneal dialysis. *BMJ Case Reports*.

